# Neural network-assisted meta-router for fiber mode and polarization demultiplexing

**DOI:** 10.1515/nanoph-2024-0338

**Published:** 2024-09-05

**Authors:** Yu Zhao, Huijiao Wang, Tian Huang, Zhiqiang Guan, Zile Li, Lei Yu, Shaohua Yu, Guoxing Zheng

**Affiliations:** Electronic Information School, and School of Microelectronics, Wuhan University, Wuhan 430072, China; Peng Cheng Laboratory, Shenzhen 518055, China; School of Physics and Technology, Wuhan University, Wuhan 430072, China; Wuhan Institute of Quantum Technology, Wuhan 430206, China.

**Keywords:** metasurface, deep learning, object recognition, space-division multiplexing

## Abstract

Advancements in computer science have propelled society into an era of data explosion, marked by a critical need for enhanced data transmission capacity, particularly in the realm of space-division multiplexing and demultiplexing devices for fiber communications. However, recently developed mode demultiplexers primarily focus on mode divisions within one dimension rather than multiple dimensions (i.e., intensity distributions and polarization states), which significantly limits their applicability in space-division multiplexing communications. In this context, we introduce a neural network-assisted meta-router to recognize intensity distributions and polarization states of optical fiber modes, achieved through a single layer of metasurface optimized via neural network techniques. Specifically, a four-mode meta-router is theoretically designed and experimentally characterized, which enables four modes, comprising two spatial modes with two polarization states, independently divided into distinct spatial regions, and successfully recognized by positions of corresponding spatial regions. Our framework provides a paradigm for fiber mode demultiplexing apparatus characterized by application compatibility, transmission capacity, and function scalability with ultra-simple design and ultra-compact device. Merging metasurfaces, neural network and mode routing, this proposed framework paves a practical pathway towards intelligent metasurface-aided optical interconnection, including applications such as fiber communication, object recognition and classification, as well as information display, processing, and encryption.

## 1 Introduction

The widespread adoption of emerging technologies, including artificial intelligence, cloud computing, big data, and edge computing, has ushered humanity into an era of data explosion, marked by a critical need for enhanced information transmission capacity [1]. This evolution has prompted advancements in fiber communication technologies, introducing few-mode fibers, multi-mode fibers [2], multi-core fibers [3], and few-mode multi-core fibers [4] for space-division multiplexing (SDM) communications [5]. Fiber mode multiplexer and demultiplexer devices are of vital importance to SDM systems, which have been extensively achieved using diverse methods, including couplers [6], waveguides [7], free-space optics [8], and photonic lanterns [9]. Currently, a SDM system in conjunction with a bulky and complex fiber-optic framework has been demonstrated, combining mode-division, polarization-division and wavelength-division multiplexing technologies [10]. To meet the demand for a highly miniaturized and integrated system, it is essential to not only consider expansion of multiplexing dimensions and parallel channels, but also design complexity and device compactness of a SDM optical fiber communication framework. However, emerging SDM systems have satisfied degrees of freedoms in transverse spatial domain but not reached acquirements of miniaturization with highly-integrated devices. Therefore, achieving divisions and recognitions in multiple dimensions comprising intensity distributions and polarization states of fiber modes through an ultra-simple design and ultra-compact devices remains a significant challenge.

Metasurfaces, planar optics patterned with subwavelength-scale nanoscatterers [11], have demonstrated ability to manipulate diverse degrees of freedom of light, including phase [12], amplitude [13], polarization [14], and frequency [15]. In the field of optics, metasurfaces have been widely utilized in realization of various optical components such as lenses [16], holograms [17], and imagers [18]. Compared to conventional optics, metasurfaces have offered both theoretical exploration and practical application as a novel platform for advancing optical fiber science and technology. Specifically, metasurfaces have delivered advantages as ultra-compact construction and wide-range integration, interfaced on fiber end-faces and embedded in various optical systems including achromatic focusing and imaging [19], flexible trapping [20], as well as structured light transforming [21]. Moreover, in the domain of mode-division multiplexing and demultiplexing, current metasurface-based devices have primarily focused on spatial mode conversion [22], [23] and mode division [24], [25] constrained to single dimension, such as intensity distributions or polarization states of modes. By simultaneously addressing intensity distributions and polarization states, metasurfaces hold the potential to achieve mode division within dual dimensions. This advancement promises to expand function channels, enhance transmission capacity, and improve integration usability of fiber communication systems.

In addition, with the assistance of deep learning methods, performances of metasurfaces have been improved to achieve stronger manipulation of light wave and richer function of optical components. Currently, deep learning methods have become pervasive optimization tools across diverse physical systems, encompassing optical, electrical, and mechanical systems [26]. Recent advancements in merging metasurfaces with neural networks have enabled various functions using single-layer [27] and multi-layer metasurfaces [28], as well as multi-channel configurations [29], providing significant advancements in system performance, data capacity and function applications [30]. Firstly, the backpropagation mechanism within neural network algorithms introduces performance metrics into loss functions, facilitating purposeful and directional gradient updates for enhanced accuracy during optimization [31]. Furthermore, neural network algorithms typically support optimization on training sets while evaluating on testing sets, and the utilization of large-volume datasets enhances computational robustness, ensuring a higher level of redundancy [32]. In addition, neural network algorithms operate on highly-general architectures, practical for a variety of functional implementations, including object recognition [33], spectral imaging [34], and lens focusing [35]. Consequently, physical systems assisted by neural networks offer triple advantages in terms of design flexibility, environment adaptability, and function scalability.

In this article, we merge metasurfaces, neural network algorithms and fiber mode routing, presenting the concept of neural network-assisted meta-router (NNMR). Note that our proposed NNMR contains only a single layer of metasurface in free space, achieving four-mode division (comprising two spatial modes with two polarization states) within two dimensions (encompassing intensity distributions and polarization states). Specifically, the proposed NNMR acts as a polarization-dependent Fourier grating, by which the phase modulation for the optical fields of the modes transmitted in the few-mode fiber (FMF) is achieved, allowing the modes to be diffracted onto distinct spatial regions for mode recognition based on the location of the diffraction spots. Considering the susceptibility of fiber mode optical fields to environmental disturbances, our framework generates datasets of slightly transformed mode fields and utilizes the backpropagation and gradient update methods within the neural network algorithm to optimize the phase profiles of the NNMR. Furthermore, to validate the practicality of the NNMR, we conduct a series of experiments where all the diffraction spots are located at the target regions in accordance with the simulation results, achieving recognition and classification of the four linearly polarized modes within dual dimensions of intensity distributions and polarization states. Looking ahead, these results pave a practical pathway for the development of this intersection method across a broad range of applications, including but not limited to intelligent metasurfaces-aided fiber communication, all-optical object recognition and classification, as well as information display, processing, and encryption.

## 2 Results

### 2.1 Concept and design principle of NNMR

**Figure 1:**
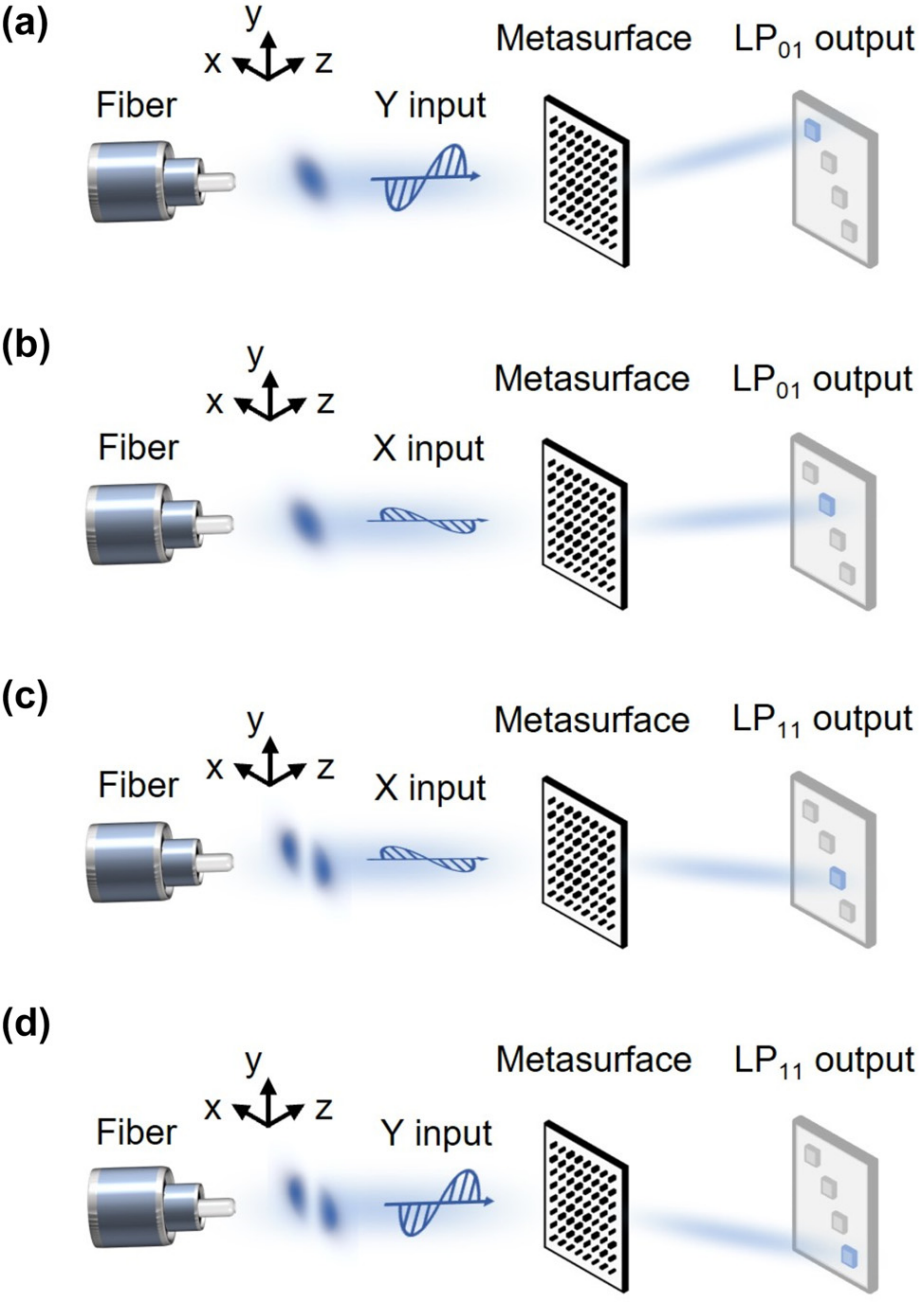
Conceptual description of NNMR and its application in four-mode recognition and space-division. (a) Recognition of the *y*-polarized LP_01_ mode. (b) Recognition of the *x*-polarized LP_01_ mode. (c) Recognition of the *x*-polarized LP_11_ mode. (d) Recognition of the *y*-polarized LP_11_ mode. The blue regions represent the target regions for each of the four modes.

Based on space division multiplexing technology in optical fiber communication, multiple spatial modes can be simultaneously and independently transmitted in a single fiber, enhancing the channel parallelism and information capacity in the spatial dimension. For instance, we chose a FMF supporting the LP_01_ and LP_11_ modes, each characterized by distinct complex amplitude distribution (see Supplementary 1 for more details about fiber configuration). Note that our framework concentrates on the division and recognition of not only the intensity distributions but also the polarization states of these modes. To achieve this, we employ a polarization-dependence metasurface, exhibiting distinct optical responses in the two orthogonal polarization directions, to spatially divide the four modes (comprising two spatial modes with two polarization states) into distinct regions. Specifically, as illustrated in Figure 1a and b, the *x*-polarized LP_01_ mode is projected onto the metasurface and diffracted onto the middle-left region of the output plane while the *y*-polarized LP_01_ mode is diffracted onto the upper-left region. Similarly, the *x*- and *y*-polarized LP_11_ modes are diffracted onto the middle-right and bottom-right regions of the output plane, respectively, as shown in Figure 1c and d.

Throughout the NNMR framework, the complex amplitude distributions of the LP_01_ and LP_11_ modes serve as the inputs and the intensity distributions of the target regions act as the outputs, as depicted in Figure 1. Additionally, a neural network algorithm is employed to model the physical system of the metasurface, optimizing the phase profiles of the metasurface to achieve the desired optical responses. By utilizing input training sets and output target regions of dual modes within identical polarization state, the neural network algorithm optimizes the phase modulations for the polarized incident modes by the metasurface. Consequently, the phase profiles of the metasurface in the *x*- and *y*-polarization directions can be determined for both the *x*- and *y*-polarized fiber modes and their corresponding target regions. Subsequently, the structural configuration of the precisely optimized metasurface is characterized by periodic nanostructures with uniform cell size but various nanobrick dimensions, resulting in polarization-dependent optical responses. As a result, a four-mode meta-router (comprising two spatial modes with two polarization states) can be realized through the polarization-dependent metasurface, dividing the four linearly polarized modes onto their respective target regions to achieve mode recognition and demultiplexing.

### 2.2 Training and simulation results of NNMR

Deep learning techniques have garnered widespread application as optimization tools in various physical systems, offering improved performance, increased capacity, and expanded functionalities. As a result, a neural network algorithm is utilized to train the NNMR, simulating light propagations through the metasurface, and optimizing the phase profiles of the metasurface accordingly.

**Figure 2:**
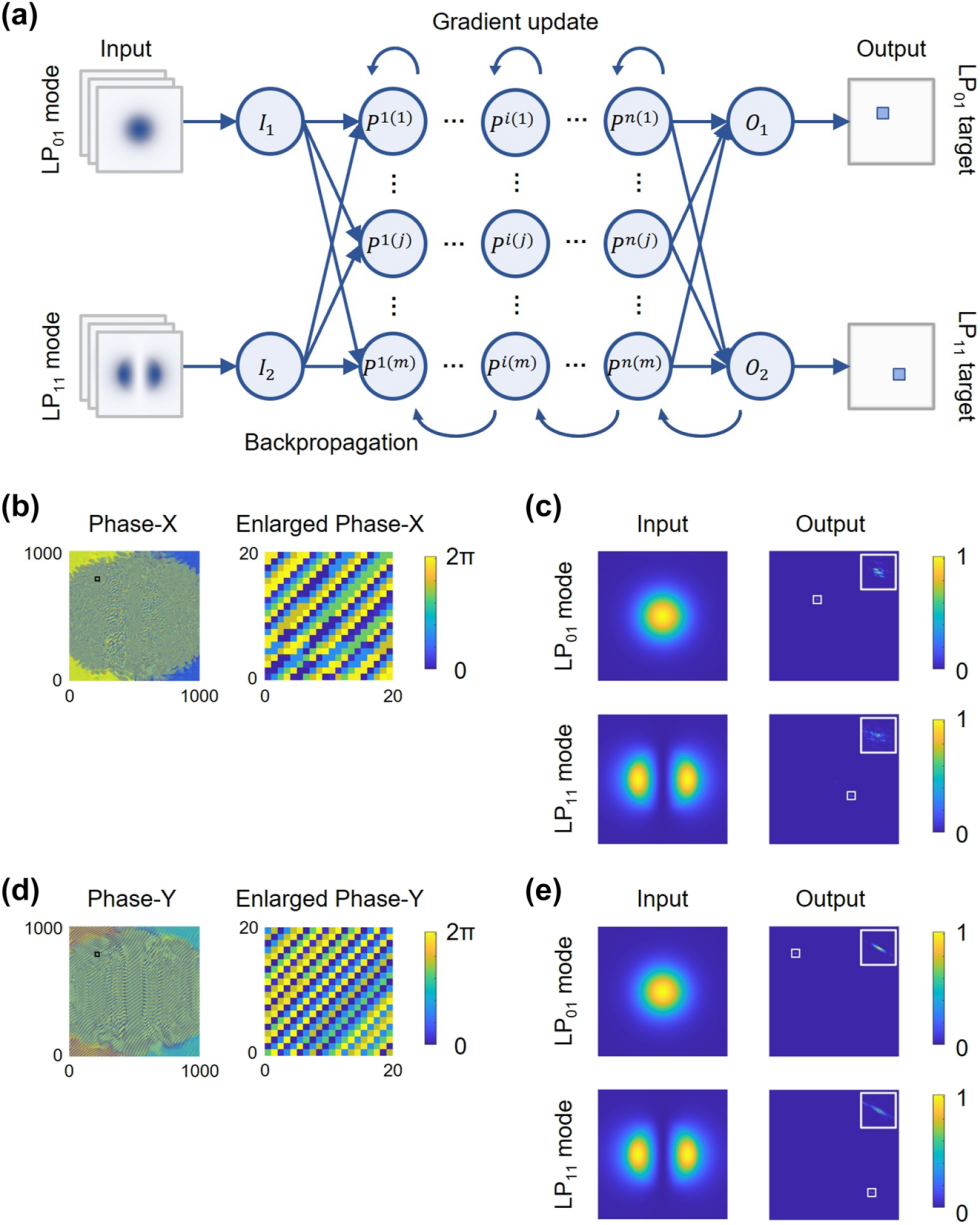
Training process of NNMR and its simulation results for four-mode recognition and space-division. (a) Training architecture of the neural network algorithm employed to optimize the metasurface. (b) Trained phase profile (1,000 × 1,000 pixels) of the metasurface in the *x*-polarization direction and its enlarged phase profile (20 × 20 pixels) in the black box of the upper-left corner. (c) Numerical simulation results of the metasurface in the *x*-polarization direction. (d) Trained phase profile (1,000 × 1,000 pixels) of the metasurface in the *y*-polarization direction and its enlarged phase profile (20 × 20 pixels) in the black box of the upper-left corner. (e) Numerical simulation results of the metasurface in the *y*-polarization direction. The white boxes highlight the target regions in the output planes and the inset images show the enlarged target regions (50 × 50 pixels). The energy of the input modes and output planes are all normalized.

The training architecture of the NNMR is illustrated in Figure 2a. The following is an example of the training process for the phase profile of the metasurface in the *x*-polarization direction. Initially, we generate the input datasets representing the transverse optical field distributions of the fiber modes, denoted as

(1) $$I\left(m,n\right)=A\left(m,n\right){\text{e}}^{\text{i}\varphi \left(m,n\right)},$$

where $A\left(m,n\right)$ and $\varphi \left(m,n\right)$ , respectively, denote the transverse amplitude and phase distribution of the modes (see Supplementary 2 for more details about datasets generation). Note that the metasurface is composed of 1,000 × 1,000 pixels, given by the areas of the mode optical fields captured by the near infrared camera (HAMAMATSU C12741-03). The ideal outputs of the architecture consist of the desirable intensity distributions in the Fourier domain (far-field) received planes for the modes, where the intensity of the target regions is set to be 1 and the background regions to 0. Specifically, the inputs for the LP_01_ and LP_11_ modes are denoted as *I*_1_, *I*_2_, respectively, and the ideal outputs are denoted as *O*_1_, *O*_2_. Additionally, the phase profile of the metasurface is represented as *P*, resulting in the phase modulation for the mode optical fields to diffract onto the target regions. Using the phase modulation by the metasurface and performing a Fourier transform in the far-field space, the actual output of the architecture can be expressed as

(2) $$O^{\text{actual}}\left(m,n\right)={\left|F\left\{A\left(m,n\right){\text{e}}^{\text{i}\left[P\left(m,n\right)+\varphi \left(m,n\right)\right]}\right\}\right|}^{2}.$$

Our objective is to minimize the disparity between the actual and ideal outputs, ensuring that the energy of each mode is precisely diffracted on its respective target region.

For this purpose, it’s crucial to minimize background noise and intermodal crosstalk for each mode as much as possible through iterations in the training process. To achieve this, we custom loss functions to assess the noise and crosstalk, denoted as Lossback and Losscross, respectively, which evaluate the errors between the actual and ideal outputs. These errors are then utilized in the backpropagation process for the gradient update of the phase profile of the metasurface. The trained results of the phase profiles in the *x*- and *y*-polarization directions are respectively illustrated in Figure 2b and d. Subsequently, using the LP_01_ and LP_11_ mode fields as inputs, we simulated the Kirchhoff diffraction through the phase modulations by the metasurface to obtain the actual outputs. The input modes and the corresponding output planes of the *x*- and *y*-polarization phase profiles are demonstrated in Figure 2c and e, respectively. It’s evident that for both the *x*- and *y*-polarization phase profiles, the actual output planes accurately locate the diffraction spots within the target regions, precisely matching the ideal output planes. Specifically, for the *x*-polarized incidence, the LP_01_ mode is diffracted onto the middle-left region, and the LP_11_ mode is diffracted onto the middle-right region in the far-field received plane. Similarly, for the *y*-polarized incidence, the LP_01_ and LP_11_ modes are, respectively, diffracted onto the upper-left and bottom-right regions in the far-field received plane.

The training and simulation results indicate that the trained metasurface theoretically achieves the division of the spatial modes and polarization states among the four modes from the FMF with high accuracy, low background noise, and minimal intermodal crosstalk. These characteristics position the trained metasurface as an exceptional candidate for a mode router, capable of accurately dividing and recognizing the modes based on both intensity distributions and polarization states.

### 2.3 Construction and modulation characterizations of NNMR

Polarization-dependent metasurface have been found widespread application for polarization multiplexing functionality within ultra-miniaturized and ultra-compact devices. In our study, we meticulously design a polarization-dependent all-silicon nanostructure for metasurface construction. This design aims to generate different phase delays for incident light in the two orthogonal polarization directions while ensuring steady transmittances. The operating wavelength of the metasurface is set to be 1,550 nm, aligning with the operating wavelength of the FMF.

**Figure 3:**
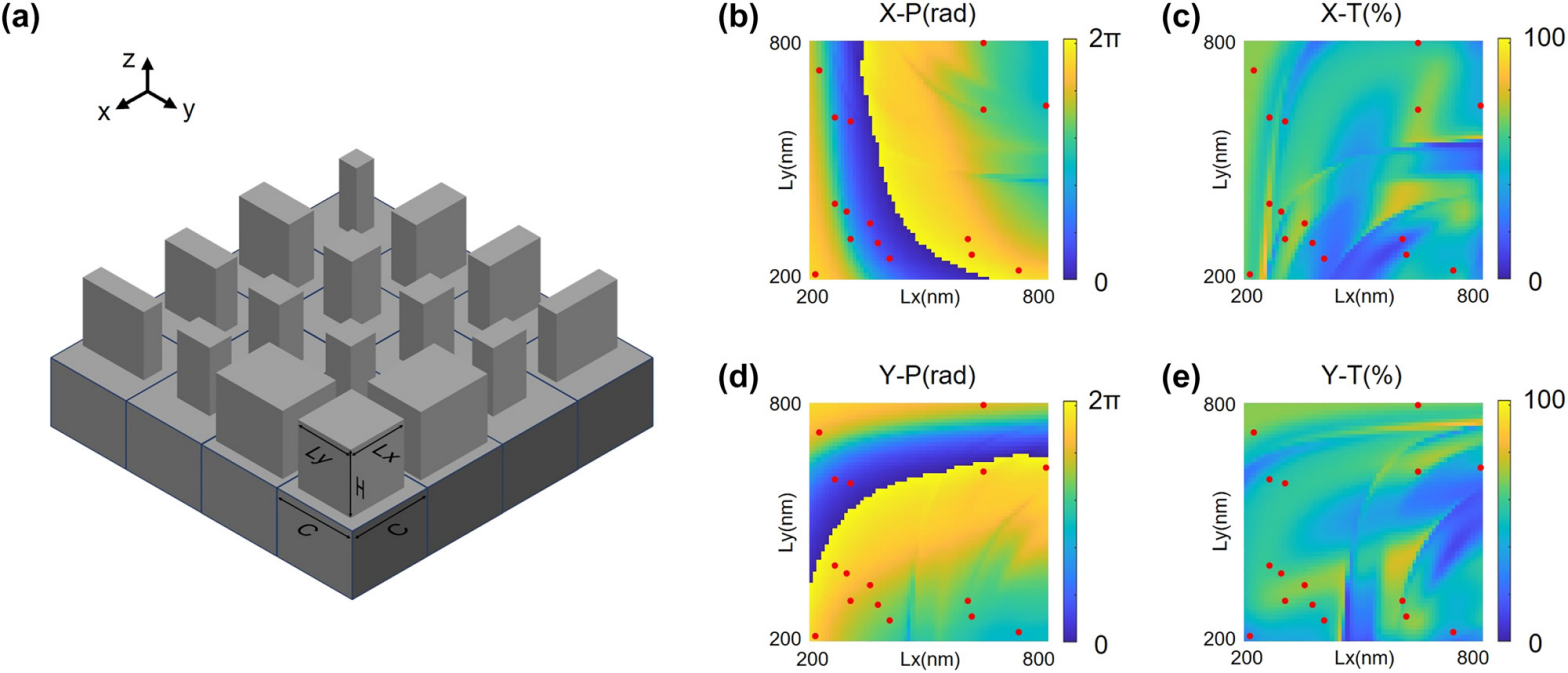
Structural construction and optical responses of NNMR for four-mode recognition. (a) Illustration of the polarization-dependent silicon nanobrick arrays laid on a silicon substrate. All the nanobricks possess an identical cell size of 900 nm and a height of 1,000 nm, but distinct lengths and widths. (b, d) Phase modulation of the transmitted light for the nanostructure unit as a function of nanobrick size *Lx* and *Ly* in the *x*- and *y*-polarization directions, respectively. (c, e) Intensity transmission of the transmitted light for the nanostructure unit as a function of nanobrick size *Lx* and *Ly* in the *x*- and *y*-polarization directions, respectively. The red spots emphasize the dimensions and characterizations of the 16 selected nanobricks.

The construction of our designed nanostructure, featuring periodic nanobrick arrays laid on the substrate with the same cell sizes *C* × *C* and height *H*, but varying lengths *Lx* and widths *Ly*, is illustrated in Figure 3a. Each nanobrick can be considered as a truncated waveguide, and nanobricks with different dimensions yield different effective refractive indices, thereby providing distinct phase delays in the *x*- and *y*-polarization directions. Consequently, by precisely tailoring the phase profile of the nanobrick arrays, they can function as effective phase modulators independently for the *x*- and *y*-polarized incidences. Leveraging this, polarization-dependent metasurfaces can achieve separate and distinct phase modulation for the *x*- and *y*-polarized incident fiber modes, which can be diffracted onto distinct target regions to recognize the polarization states of the modes.

As mentioned above, the phase modulation and intensity transmission of each nanostructure unit are determined by the length and width of the nanobrick. Therefore, the COMSOL Multiphysics software is used to simulate and calculate the nanostructure unit. We fixed the cell sizes and height of the nanobrick, and then scanned the length and width of the nanobrick, finally calculating the phase and intensity modulation for the transmitted light. The fixed parameters include nanobrick cell sizes of 900 nm × 900 nm and a height of 1,000 nm, while the variable parameters consist of the length and width of the nanobrick, scanned within the range from 200 nm to 800 nm. The phase and transmittance variations as functions of the variable parameters in the *x*-polarization direction are respectively shown in Figure 3b and c, while those in the *y*-polarization direction are shown in Figure 3d and e. In this context, X–P and X–T represent the phase delays and transmittances for the *x*-polarized incidence, respectively, while Y–P and Y–T represent the phase delays and transmittances for the *y*-polarized incidence, respectively. Evidently, the phase variations in both the *x*- and *y*-polarization directions cover the range from 0 to 2*π*, ensuring the potential selection of desirable nanobrick sizes from the scanning parameters. For the 4-step phase modulation, we carefully select 16 different nanobricks capable of providing phase delay combinations of 0, *π*/2, *π*, and 3*π*/2 in the *x*- and *y*-polarization directions. The structural dimensions and optical characterizations of the 16 selected nanobricks are detailed in Table 1 and emphasized by the red spots in Figure 3b–e.

**Table 1:** Sample table with header.

No.	1	2	3	4	5	6	7	8	9	10	11	12	13	14	15	16
*Lx* (nm)	210	600	610	730	300	350	370	400	260	290	300	800	220	260	640	640
*Ly* (nm)	210	300	260	220	600	340	290	250	610	370	300	640	730	390	800	630
X–P (rad)	0.67	0.68	0.89	0.67	2.17	2.14	2.20	2.18	3.80	3.78	4.33	4.31	5.52	5.30	5.31	5.37
X–T (%)	67	61	72	57	54	51	53	53	57	51	52	53	52	50	51	51
Y–P (rad)	0.67	2.17	3.80	5.52	0.68	2.27	3.78	5.61	0.89	2.20	4.33	5.31	0.67	2.20	4.31	5.39
Y–T (%)	67	54	57	52	61	52	51	50	72	53	52	51	57	50	53	52

The established construction parameters and simulation results provide evidence that the polarization-dependent metasurface, constructed with these meticulously arranged nanobricks, can independently modulate incidences in the two orthogonal polarization states. Consequently, by arranging these nanobrick arrays based on the trained phase profiles in the *x*- and *y*-polarization directions, the resulting metasurface can exactly perform the desirable phase modulation of the *x*- and *y*-polarized incidences, diffracting the modes onto the designed target regions and achieving the recognition and demultiplexing of these modes.

### 2.4 Verification and experiment results of NNMR

**Figure 4:**
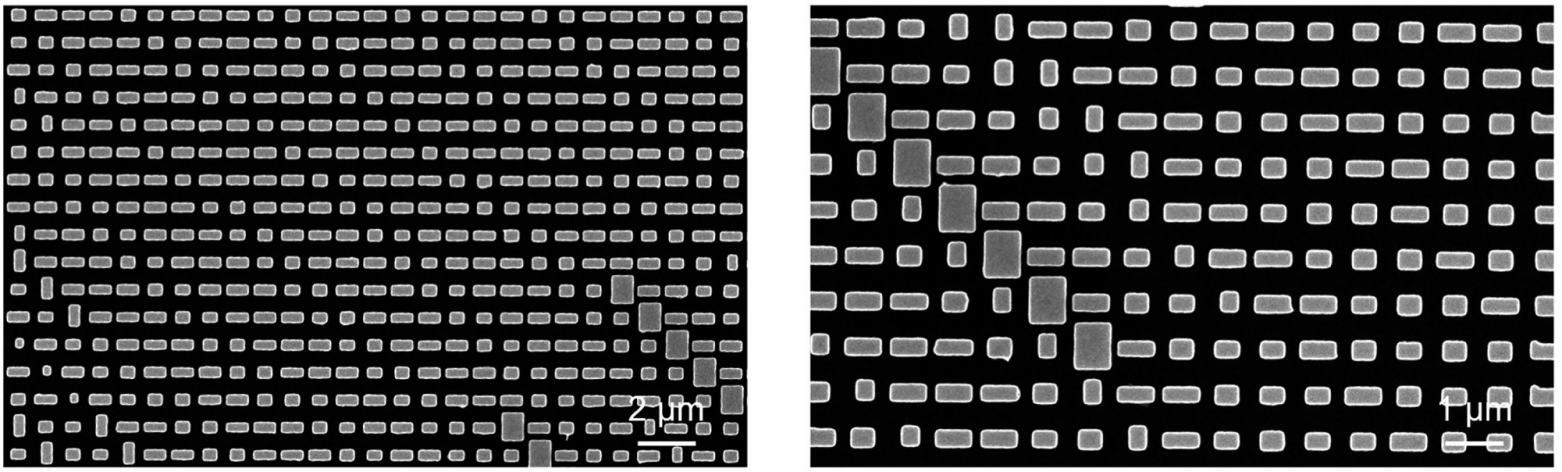
Partial scanning electron microscope (SEM) images of the fabricated metasurface.

To verify the feasibility of our concept, we fabricated a metasurface and established an optical path in free space to characterize its optical responds for a mode router (see Supplementary 3 for details about the metasurface fabrication). Figure 4 shows the partial scanning electron microscope (SEM) image of the metasurface sample.

**Figure 5:**
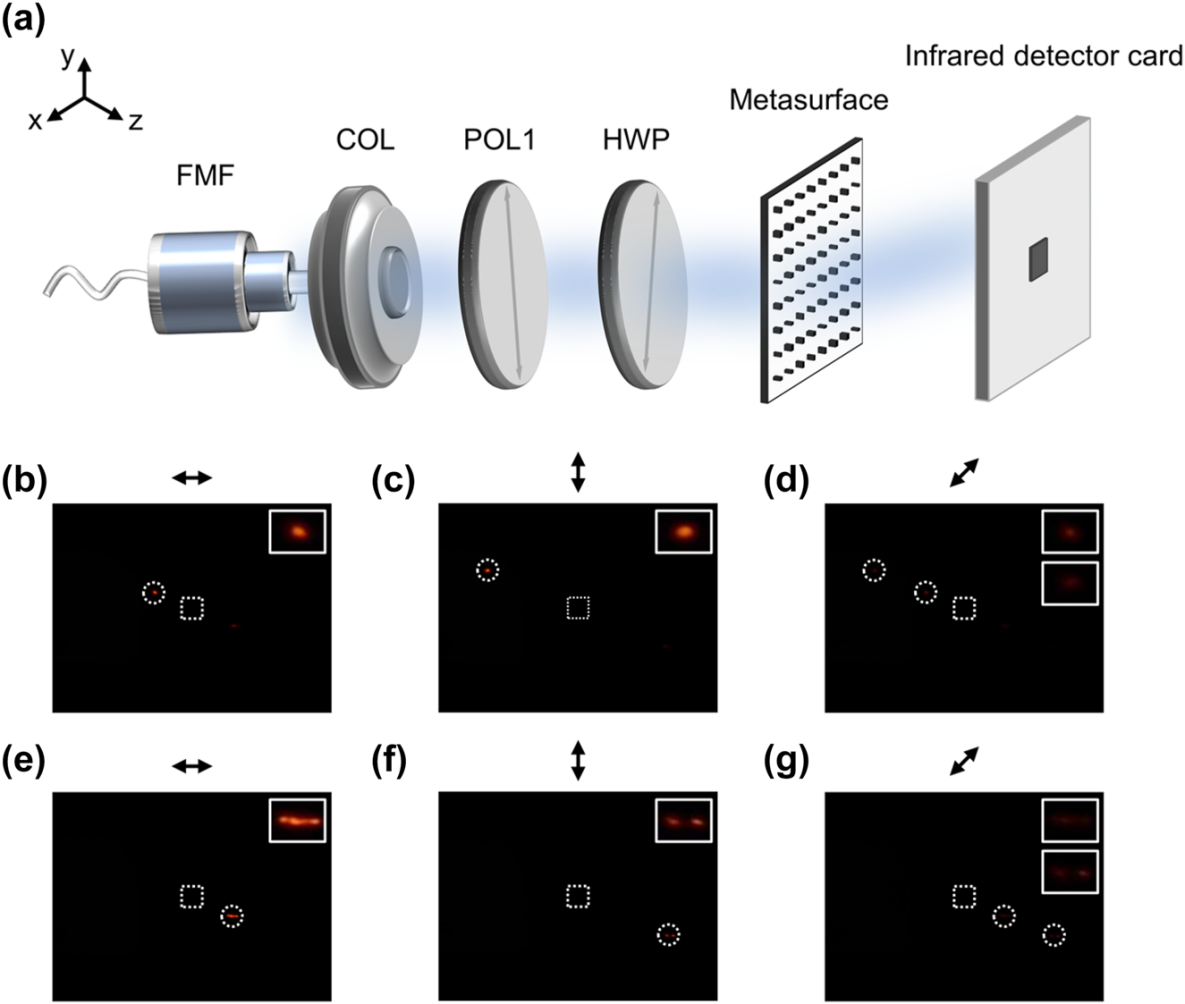
Experimental arrangement and results of NNMR for four-mode recognition. (a) Optic setups to receive the diffraction patterns from the metasurface under the polarized mode incidences. (b–d) Diffraction spots in the diffraction patterns under the *x*-polarized, *y*-polarized and 45° polarized LP_01_ mode incidence, respectively. (e–g) Diffraction spots in the diffraction patterns under the *x*-polarized, *y*-polarized and 45° polarized LP_11_ mode incidence, respectively. The dotted circles highlight the diffraction spots of the polarized modes, and the inset images show the enlarged diffraction spots. The dotted boxes delineate the edge of the black cardstock in the middle of the infrared detector card. All experiments were conducted at an operating wavelength of 1,550 nm.

The experimental arrangement for functional verification of our designed NNMR is depicted in Figure 5a. A tuneable laser (THORLABS TLX1) served as the light source for a FMF operating at 1,550 nm. The FMF supported the LP_01_ and LP_11_ modes, and a collimating lens (COL, THORLABS PAF2-2C) was used to collimate the transverse optical fields of these modes. Subsequently, the optical fields passed through a polarizer (POL1, LBTEK FLP25-NIR-M) to maximize power, and then through a half-wave plate (HWP, LBTEK HWP20-1550B) to adjust the polarization states to *x*-polarization, *y*-polarization, or 45° polarization. The polarization directions of POL1 and HWP were calibrated in free space beforehand (see Supplementary 4 for details about the polarization calibration). The *x*-polarized, *y*-polarized or 45° polarized mode was directed onto the metasurface and then diffracted onto a fixed region in the far-field received plane. An infrared detector card (THORLABS VRC4), featuring a black cardstock (both length and width of 2 mm) to block zero-order light, was positioned 6 mm along the optical axis from the metasurface.

The diffraction patterns of all the modes in the far-field received plane were detected by the infrared detector card and captured by a commercial camera (Nikon D5100). Through polarization control (*x*-polarization, *y*-polarization and 45° polarization) and mode conversion (LP_01_ and LP_11_ mode), the location of the diffraction spot in the diffraction pattern was transformed, as illustrated in Figure 5b–g. Notably, under the *x*- and *y*-polarized LP_01_ mode incidence, two diffraction spots respectively located in the middle-left and upper-left regions could be easily switched, and under the *x*- and *y*-polarized LP_11_ mode incidence, diffraction spots respectively located in the middle-right and bottom-right regions could be easily switched. When the incident mode was 45° polarized, corresponding diffraction spots in the middle-left and upper-left regions were simultaneously observed under the LP_01_ mode incidence, and diffraction spots in the middle-right and bottom-right regions were observed under the LP_11_ mode incidence. Additionally, another infrared detector board (LBTEK IRDC1-200S-M-SP230614) was positioned 5 cm from the metasurface to detect the diffraction patterns at a longer observing distance. The measured distances between the diffraction spots and the zero-order spots for the LP_01_ and LP_11_ modes in the *x*-polarization state were both 3.2 cm, corresponding to diffraction angles of 32.6°. For the *y*-polarization state, the measured distances were 7.2 and 7.1 cm for the LP_01_ and LP_11_ modes, respectively, corresponding to diffraction angles of 55.2 and 54.8°. These results were consistent well with the theoretical simulation values, which predicted distances of 3.1, 3.2, 7.2, and 7.1 cm, and angels of 32.0, 32.4, 55.3 and 54.9°.

To evaluate the performance of our meta-router, we introduced quantitative indicators including insertion loss, modal/polarization crosstalk and mode purity. Specifically, the power of these polarized mode incidences and their diffraction spots were measured. The measured insertion loss were, respectively, −16.0 and −15.9 dB for the *x*-polarized LP_01_ and LP_11_ modes, while −17.0 and −16.8 dB for the *y*-polarized LP_01_ and LP_11_ modes. The measured model crosstalk were respective −3.3 and −9.4 dB for the *x*-polarized LP_01_ and LP_11_ modes, while −4.0 and −11.9 dB for the *y*-polarized LP_01_ and LP_11_ modes. The measured polarization crosstalk were, respectively, −10.8 and −8.0 dB for the *x*- and *y*-polarized LP_01_ modes, while −12.3 and −13.8 dB for the *x*- and *y*-polarized LP_11_ modes. As for the mode purity of the diffraction spots, we fitted the captured spot profiles and the simulated mode fields and calculated their correlation coefficients. The coefficients of the spot profiles were respectively 0.9723 and 0.9703 under *x*- and *y*-polarized LP_01_ mode incidences, while 0.6789 and 0.7448 under *x*- and *y*-polarized LP_11_ mode incidences. Additionally, we measured the power variations of the diffraction spots when misalignments between the mode incidences and the metasurface occurred in free space (see Supplementary 5 for more details about misalignment sensitivity).

The experimental results demonstrate that our designed metasurface successfully achieves spatial division of the four modes from the FMF in two dimensions, encompassing intensity distributions and polarization states, with high accuracy using a single-cell unit and single-layer construction. Consequently, our proposed NNMR framework provides high-quality mode recognition and classification in dual dimensions through an ultra-simple design and ultra-compact device.

## 3 Discussion

We have incorporated metasurfaces and deep learning methods into optical fiber mode routing, enabling recognition across two dimensions: intensity distributions and polarization states of spatial modes. This pioneering approach opens a novel avenue for optical fiber mode demultiplexing in terms of new dimensions, methodologies, and applications. From a device integration standpoint, nanoscale metasurface facilitates ultra-miniaturization and ultra-compactness of multimode recognition systems, replacing bulky mode multiplexers and demultiplexers. Moreover, leveraging the multidimensional modulation and multichannel transmission of light by metasurfaces, such as amplitude, phase, polarization, and orbital angular momentum, multiplexing dimensions and parallel channels of systems can be extended. For instance, an optimization process is replicated for four spatial modes (the LP_01_, LP_11_, LP_02_, and LP_21_ modes) and the simulation results indicates that the trained metasurface enables spatial divisions for these four modes (see Supplementary 6 for more details about function scalability). In terms of methodology, use of neural network algorithms for metasurface optimization enhances performance metrics and data capacity, deepening integration of deep learning methods into metasurface device design. Crucially, our framework enables accurate recognition of both spatial intensity distributions and polarization states of fiber modes, advancing classification of fiber mode within dual dimensions in the field of metamaterials. Moreover, an optical communication platform based on our meta-router can be established, which contains mode route, signal transmission and reception blocks, to facilitate mode- and polarization-division multiplexing communications. Consequently, ultra-compact, multi-function, and high-quality neural metasurface platforms can be developed to achieve a variety of communication systems for data transmission and information classification. This development paves a practical pathway towards diverse emerging applications, including space-division multiplexing communication, object recognition and classification, as well as information display, processing, and encryption.

## Supplementary Material

Supplementary Material Details
