# Supplementary material for: Neural network-assisted meta-router for fiber mode and polarization demultiplexing
Source: Nanophotonics. 2024 Sep 5;13(22):4181–9. doi: 10.1515/nanoph-2024-0338 (PMC11501066; doi:10.1515/nanoph-2024-0338)
Supplement: Supplementary file 1 — Supplementary Material Details [file j_nanoph-2024-0338_suppl_001.docx]

Supplemantary

**Neural network-assisted meta-router for fiber mode and polarization demultiplexing**

*Yu Zhao, Huijiao Wang, Tian Huang, Zhiqiang Guan, Zile Li, Lei Yu, Shaohua Yu, Guoxing Zheng*

**1. Few-mode fiber configuration**

A fused-type fiber mode couple (FFMC) is used for mode converting and mode coupling, as shown in Fig. S1. The FFMC is composed of single-mode fiber (SMF) and few-mode fiber (FMF) which are fused by traveling flame along the longitudinal direction, enabling the light coupled from one piece of fiber to another piece. The FMF is fabricated by Fiberhome Corporation, which supports the LP_01_ mode and LP_11_ modes. Specifically, the LP_01_ mode transmitted through the FMF maintains LP_01_ mode in fused area and the output mode field form the FMF is still LP_01_ mode. In addition, when the LP_01_ mode is launched into the SMF port, the mode coupling occurs, and part of energy transform into the LP_11_ mode, and the output mode fields contain LP_01_ mode from the SMF and LP_11_ mode from the FMF. The diameter, cladding diameter and coupling length are carefully designed to avoid the converted LP_11_ mode in the FMF converting back to the SMF. In simulation, the cladding diameter and index of the SMF and FMF are 125 *μ*m and 1.444 and the core diameter of the two fibers are 9 *μ*m and 13.5 *μ*m, respectively. The above parameters are used to obtain the standard transverse amplitude distribution of the mode fields in data collection.

**
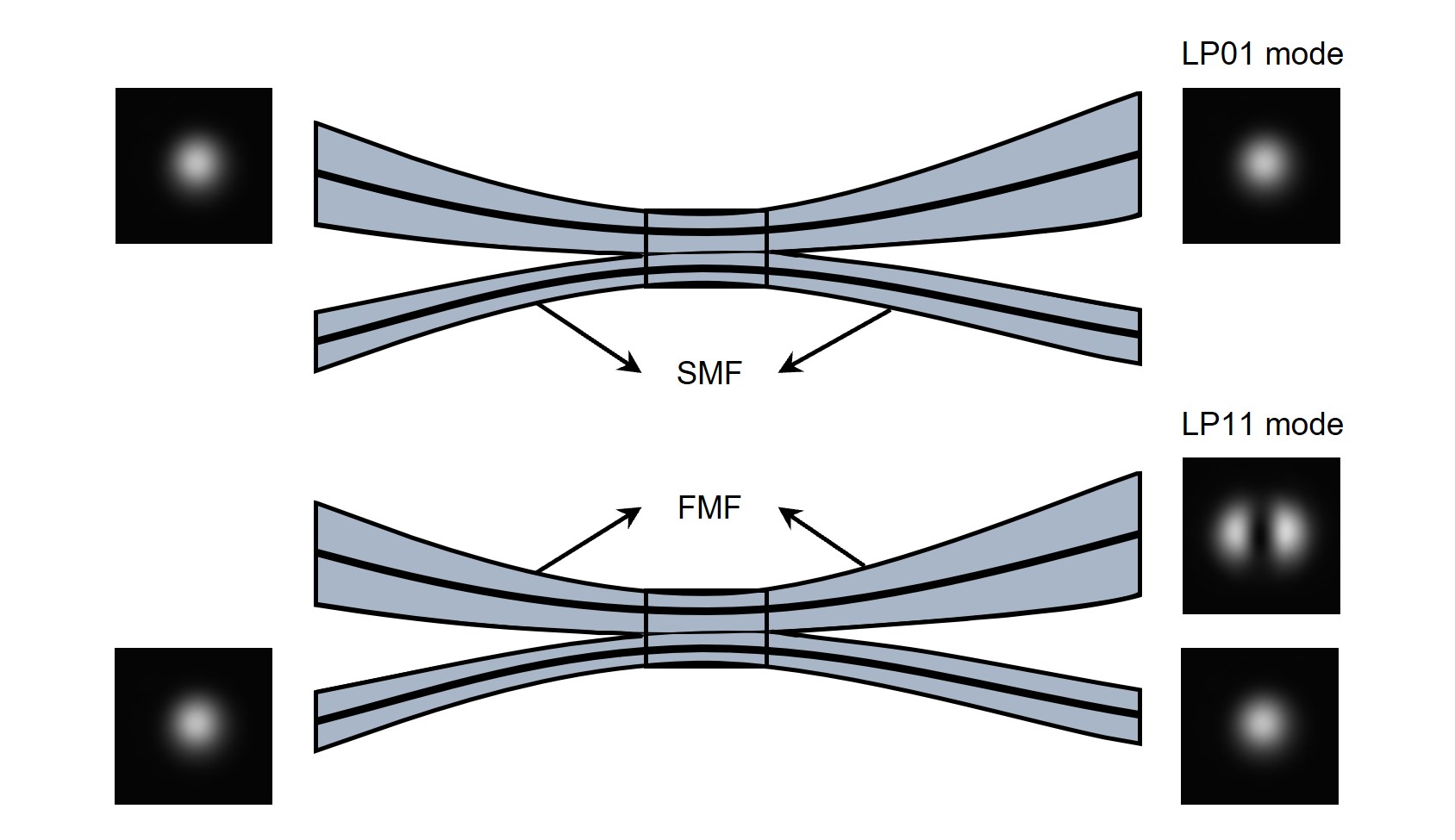
**

**Fig. S1:** Model and characterization of the FFMC. The field distributions for the LP_01_ and LP_11_ modes from the FMF are captured by the infrared camera.

**2. Data collection for neural network training**

Initially, relying on the theoretical dimension parameters of the FMF, we generate reference field distributions of the LP_01_ and LP_11_ modes. Subsequently, we fit the reference field distributions to actual field distributions captured by the infrared camera to obtain the standard field distribution, exhibiting a correlation coefficient of 0.9851 and 0.9737 in comparison with the actual field distribution, respectively for the LP_01_ and LP_11_ modes.

Then based on the standard field distributions, we introduce variable parameters, adjustable within experimentally controllable ranges, to generate field distribution datasets. As for position translation, considering the unequal areas of the LP_01_ and LP_11_ mode standard field distributions, we set a scale for axial shift of the LP_01_ mode in the positive and negative directions of the x and y axes to be 8.6 µm with a resolution of 1.72 µm, and a scale of 10.75 µm with a resolution of 2.15 µm for the LP_11_ mode. Addressing the planar rotation, we establish a rotation scale of 2 degrees for both clockwise and counterclockwise directions for the two modes, with a resolution of 1 degree. For the areal zoom, we set a reduction scale of 0.9 and an enlargement scale of 1.1 both with a resolution of 0.01 for the two modes. In Fig. S2, within the datasets, the original distributions without any transformation for the two modes are shown, and transformed distributions with an overlay transformation containing axial shift, planar rotation, and areal zoom, all of which are at a maximum scale are also displayed.


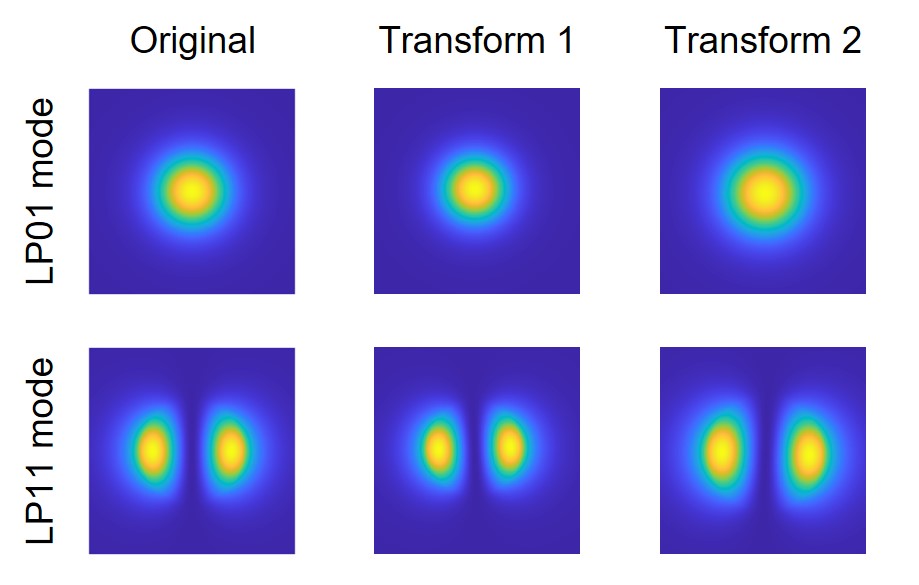


**Fig. S2:** Original and maximally transformed distributions for the LP_01_ and LP_11_ modes in datasets.

**3. Sample fabrication**

Our proposed NNMR contained a single layer of metasurface, featuring an all-dielectric structure made of monocrystalline silicon. Firstly, a PMMA photoresist with a concentration of 1:1 was spin-coated onto the sample, which underwent an exposure process through an electron beam exposure machine (eLINE Plus) based on the designed construction of the nanobricks. Following this, a 25-nm-thick film of Cr was thermally evaporated after the development and fixation process. The Cr in the unexposed area was removed through a lift-off process, forming the Cr film consistent with the designed construction of nanobricks on the surface. Finally, etching was conducted using an inductively coupled plasma etcher (PlasmaPro 100Cobra 300) with an etching depth of 1000 nm. And a corrosive is employed to eliminate the remaining Cr, obtaining the final metasurface.

**4. Polarization calibration in experiments**

In order to verify the function of the metasurface on the separation and recognition for linearly polarized modes, it is necessary to adjust the polarization states of the modes using a polarizer and a half-waveplate before the metasurface. Therefore, the adjustment effect of both the polarizers (POLs, LBTEK FLP25-NIR-M) and half-wave plate (HWP, LBTEK HWP20-1550B) on the modes was spatially calibrated.

A tunable laser source (THORLABS TLX1) provided light for a FMF at an operating wavelength of 1550 nm. As shown in Fig. S4a, light was transmitted into the FMF and then the mode optical field from the FMF was collimated by a collimating lens (THORLABS PAF2-2C). The collimated mode was adjusted to circularly-polarized light by a polarizer (POL1, FLP25-NIR-M) and a quarter-wave plate (QWP, LBTEK QWP20-1550B), and then adjusted to standard linearly-polarized light through another polarizer (POL2, FLP25-NIR-M). Subsequently, the x-polarized component of the standard linearly-polarized light was transmitted and y-polarized component was reflected through a polarizing beam splitter (PBS, LBTEK MPBS644). An infrared sensor (THORLABS S122C) was fixed along the optical axes from the PBS for the measurement of the power of the transmitted x-polarized component with a digital console (THORLABS PM122D). Specifically, the polarization state of the mode projected to the PBS was changed by rotating the POL2, when the digital console shows the maximum, the transmitted mode from the POL2 was closest to the x-polarization, similarly when the digital console shows the minimum, the transmitted mode was closest to the y-polarization.

As for the POL1, we calibrated it with POL2. As shown in Fig. S4b, the collimated mode was transmitted into the POL1 and POL2, and then received by the sensor. Firstly, the rotation angle of the POL2 was fixed to ensure that only x-polarized mode was transmitted and received by the sensor. And the polarization state of the mode was changed by rotating the POL1, when the digital console shows the maximum, the transmitted mode from the POL1 was closest to the x-polarization, similarly when the digital console shows the minimum, the transmitted mode was closest to the y-polarization.

Additionally, for the calibration of the HWP, we considered the optical module containing the POL1 and the HWP for the experiments in the main text, as shown in Fig. S4c. Firstly, the rotation angle of the POL2 was fixed to ensure that only x-polarized mode was transmitted and received by the sensor. And then, for the LP_01_ mode incidence, we rotated the POL1 and fixed it when the transmitted energy from the POL1 reached the maximum for the higher efficiency. Subsequently, the HWP was rotated to change the polarization state of the mode, when the digital console shows the maximum, the transmitted mode from the HWP was closest to the x-polarization, similarly when the digital console shows the minimum, the transmitted mode was closest to the y-polarization. Likewise, for the LP_11_ mode incidence, rotating the POL1 and the HWP to obtain the x- and y-polarized mode.

As a result, the rotation angle of the POL1, POL2 and the module containing the POL1 and the HWP to obtain the x- and y-polarized mode for the LP_01_ and LP_11_ modes was marked, respectively.


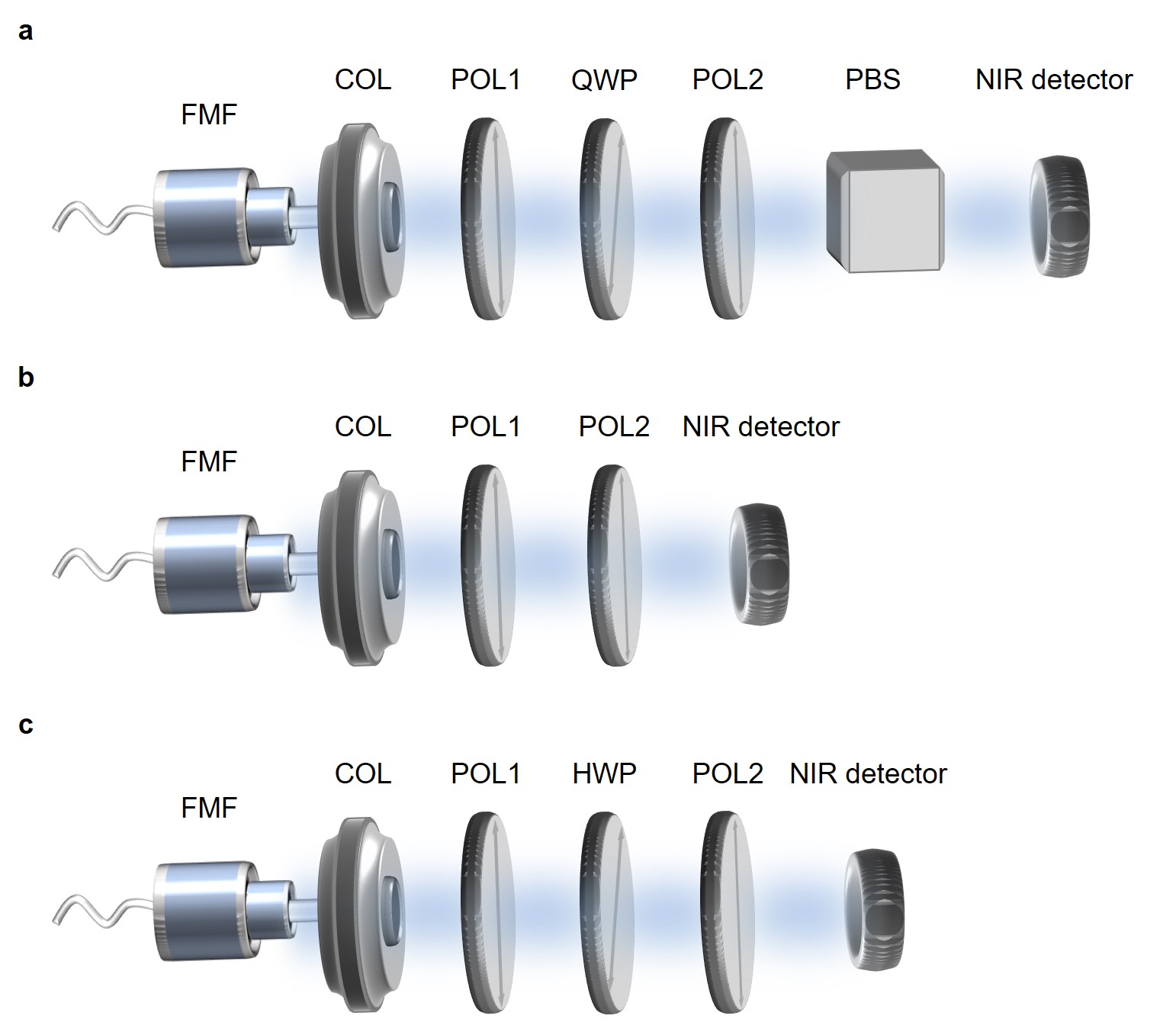


**Fig. S3:** Experimental arrangements for polarization calibration. a) Experimental optical path for calibration of POL2. b) Experimental optical path for calibration of POL1. c) Experimental optical path for calibration of the module containing POL1 and HWP.

**5.** **Misalignment sensitivity of the metasurface in experiments**

To assess the misalignment sensitivity of the metasurface in our experiments, we set the original position of the metasurface, where the diffraction spots is depicted in Fig. 5b-g. Subsequently, we shifted the metasurface along the x- and y-axes causing misalignment between the mode incidences and the metasurface in free space. The measured power variations of the diffraction spots (differences in the energy of the diffraction spots between the original and translated positions of the optical path) for the x- and y-polarized LP01 and LP11 modes is illustrated in Fig. S4. The units for translations of the metasurface were set as 50 and 100 μm along the x- and y-axes, respectively. Within the range of one unit along the positive and negative direction, the power variations of the diffraction spots remained higher than -1 dB for the four modes in both the x- and y-axial translations. Moreover, within the range of two units, the power variations for these modes were higher than -2 dB along these translations.


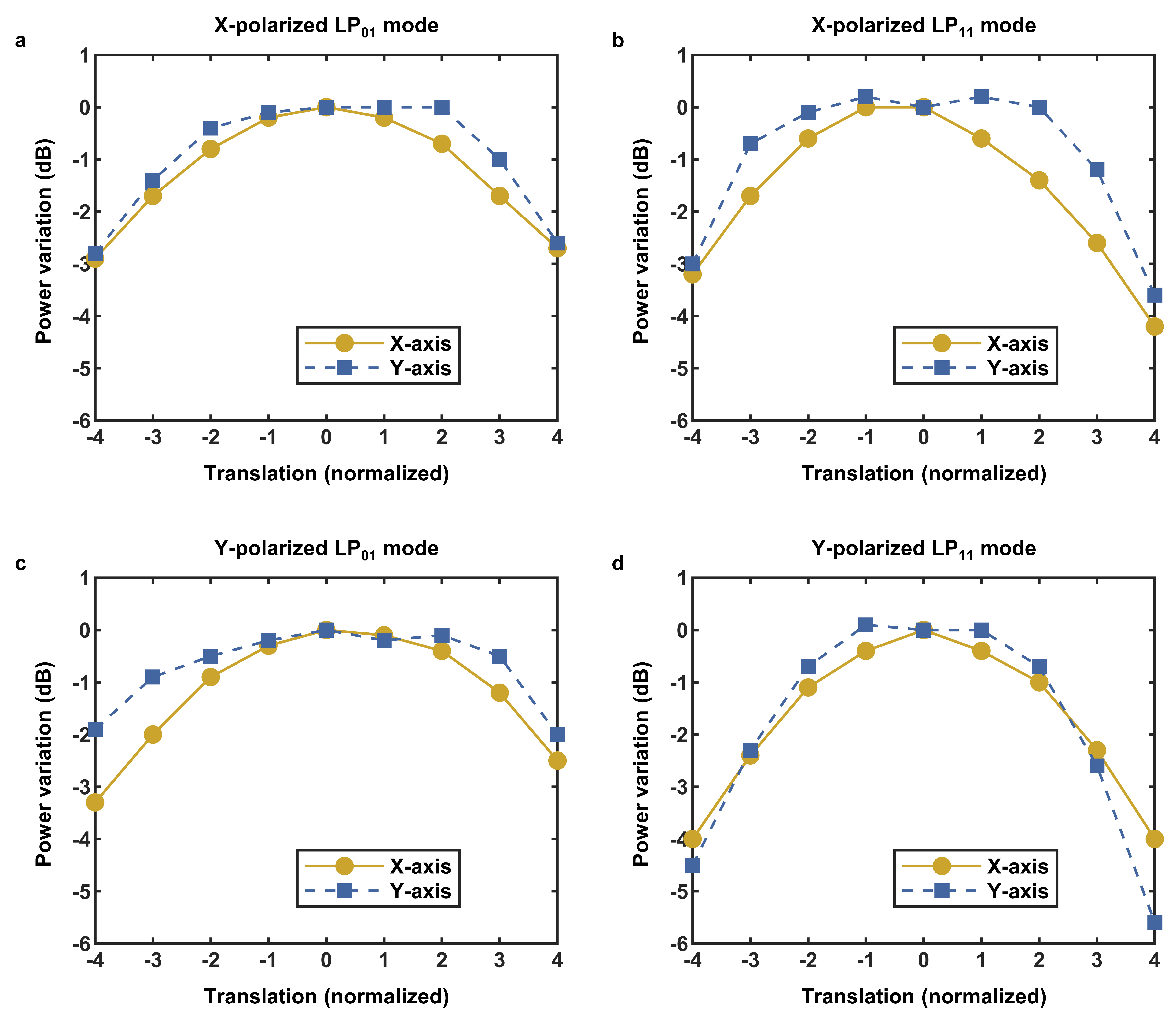


**Fig. S4:** Misalignment assessments of the metasurface. a) Power variations of the diffraction spot versus position translations for the x-polarized LP_01_ mode. b) Power variations of the diffraction spot versus position translations for the x-polarized LP_11_ mode. c) Power variations of the diffraction spot versus position translations for the y-polarized LP_01_ mode. d) Power variations of the diffraction spot versus position translations for the y-polarized LP_11_ mode.

**6. Function scalability for the eight-mode meta-router**

We theoretically designed and experimentally demonstrated a framework of four-mode meta-router trained by a neural network architecture, allowing the modes to be diffracted onto distinct spatial regions for mode recognition based on the location of the diffraction spots. The scalability for the parallel channels within the meta-router for a larger number of distinguished modes is theoretically achieved under the computation capacity of the neural network architecture and the modulation ability of the metasurface within a single layer.

For instance, the optimization process is replicated for four spatial modes (the LP01, LP11, LP02, and LP21 modes) to achieve divisions for more modes. The trained phase profile of the metasurface and numerical simulation results of the Kirchhoff diffraction is shown in the Fig. S5. Evidently, the trained metasurface enables spatial divisions for all the four modes. Therefore, two phase profiles which are trained employing two sets of output targets can be utilized for the x- and y-polarized modes to achieve eight-mode divisions and recognitions. Essentially, besides the optimization process, the nanostructure design, fabrication process, and system configuration can be generally replicated for the multi-mode NNMR.


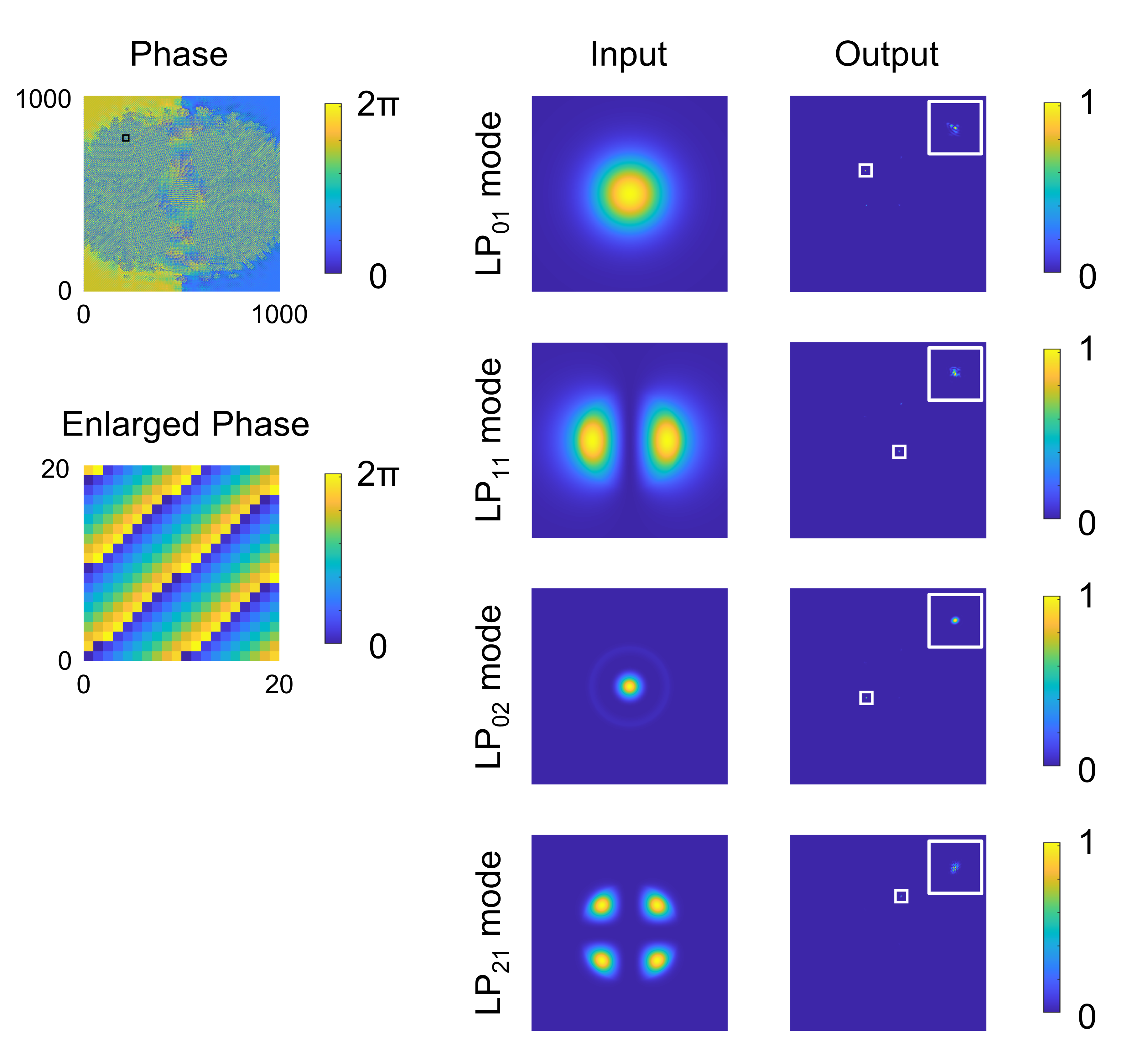


**Fig. S5:** Optimization and simulation results of the metasurface for eight-mode recognition. a) Trained phase profile (1000 × 1000 pixels) of the metasurface and its enlarged phase profile (20 × 20 pixels) in the black box of the upper-left corner. b) Numerical simulation results of the metasurface. For output targets, the white boxes highlight the target regions in the output planes and the inset images show the enlarged target regions (50 × 50 pixels). The energy of the input modes and output planes are all normalized.
